# Mairá-Potato (*Casimirella* sp.): Botanical, Food, Pharmacological, and Phytochemical Aspects

**DOI:** 10.3390/molecules28166069

**Published:** 2023-08-15

**Authors:** Danusa Silva da Costa, Lucely Nogueira dos Santos, Nelson Rosa Ferreira, Katiuchia Pereira Takeuchi, Alessandra Santos Lopes

**Affiliations:** 1 LABIOTEC/FEA (Biotechnological Process Laboratory/Faculty of Food Engineering), ITEC (Institute of Technology), UFPA (Federal University of Pará), Rua Augusto Corrêa S/N, Guamá, Belém 66075-900, PA, Brazil; lucelynogueira@gmail.com (L.N.d.S.); nelson.ufpa@gmail.com (N.R.F.); alessalopes@ufpa.br (A.S.L.); 2 Department of Food and Nutrition, Faculty of Nutrition, UFMT (Federal University of Mato Grosso), Cuiabá 78060-900, MT, Brazil; katiuchia.takeuchi@gmail.com

**Keywords:** new starch, non-conventional starch, review, chemistry structure, application hints

## Abstract

Millions of people in the world live in food insecurity, so identifying a tuber with characteristics capable of meeting the demand for food and also identifying active compounds that can be used to minimize harm to human health is of great value. The aim was to carry out a review based on systematic review tools and the main objective was to seek information on botanical, food, pharmacological, and phytochemical aspects of *Casimirella* sp. and propose possible applications. This review showed papers that addressed botanical, food, pharmacological, and phytochemical aspects of the Mairá-potato and presented suggestions for using this tuber allied to the information described in the works found in the Google Academic, Scielo, Science Direct, Scopus, PubMed, and Web of Science databases. This review synthesized knowledge about the Mairá-potato that can contribute to the direction of further research on the suggested technological applications, both on the use of this tuber as a polymeric material and its use as biomaterial, encapsulation, bioactive use, and 3D printing, because this work collected information about this non-conventional food plant (PANC) that shows great potential for use in various areas of study.

## 1. Introduction

One person out of nine does not have enough food for a healthy life. Around 805 million people worldwide are food insecure according to the Food and Agriculture Organization of the United Nations (FAO). This insecurity is due to the poorer classes being unable to obtain enough food to meet an adequate diet [1]. But, in 2022, the FAO reported that in 2021 about 828 million people in the world, approximately 10.5% of the world’s population, faced hunger and estimated that by 2030 almost 670 million people are expected to remain undernourished [2]. The discussion around the inability to meet human food demand is increasingly present due to population projections that indicate increased consumption, growth of cities, and restrictions on land exploitation [3].

Root and tuber crops are tropical countries’ second largest cultivated species after cereals. They occupy a privileged position in food security because of their high carbohydrate content and calorific value [4]. They can contain various medicinally important bioactive principles found in different parts such as tubers, stems, and leaves; furthermore, tubers serve as different carbohydrate depots [5].

Identifying a plant with constituents that promote health benefits is of great interest. Foods of plant origin have a wide variety of non-nutritive phytochemical compounds. They are synthesized as secondary metabolites and serve various ecological functions in plants [6]. Tubers are significant sources of several compounds, such as saponins, phenolic compounds, glycoalkaloids, phytic acids, carotenoids, and ascorbic acid. Various bioactivities, such as anti-oxidant, immuno-modulatory, anti-microbial, anti-diabetic, anti-obesity, and hypo-cholesterolemic, are reported for tubers and roots [7].

Brazil’s incredible biodiversity includes the Mairá-potato, an unconventional food plant [8]. Non-conventional food plants (PANC) have one or more edible parts, either wild or cultivated or native or exotic, which are not part of the daily diet of the population [9]. To consume a PANC, one must respect its characteristics and preparation methods to safely obtain its properties [10].

Moreover, when we think of food demand and species such as the Mairá-potato (that is not very well known), it could be considered an option not yet explored in intensive production, which, if further studied, could contribute to food security, since this tuber can weigh over a hundred kilograms according to reports from [11] and [12]. It also has various terpenoids in its composition [13]. This makes it a promising species for biomolecule extraction and food use. The aim was to carry out a review based on systematic review tools focusing mainly on searching for information on botanical, dietary, pharmacological, and phytochemical aspects of *Casimirella* sp. and proposing possible applications.

## 2. Results and Discussion

The survey returned 19 papers on botanical, food, pharmacological, and phytochemical aspects of *Casimirella* sp. (16 articles and 3 theses or dissertations) (Table 1).

### 2.1. Origin and Distribution of the Mairá-Potato 

The Mairá-potato belongs to a genus known as Neotropical because of its distribution in South and Central America; it is composed of seven species [18]. These species’ distribution and endemic occurrence in the Brazilian Amazon Forest (in the Amazonas and Pará) has been recorded [18,31]. Two species have been found in the Amazon Forest (*Casimirella ampla* and *Casimirella rupestris*), while the others are restricted to Brazil in the Cerrado and Pantanal [31]. According to Howard [18] and Amorim [31], four species occur in Brazilian territory. 

The genus *Humirianthera* comprises only two feculent species [32], *H. ampla* (Miers) Baehni and *H. rupestris* Ducke, which are easily distinguished from all other species of the Icacinaceae family by having an enormously enlarged connective, which resembles the tip of a spear, extends like a limb in the shape of a triangle, and has at the base two wing-like anther sacs [33]. Howard [18] cited that the genus *Humirianthera* is accepted as a synonym of *Casimirella*, which will be adopted henceforth.

### 2.2. Taxonomic Classification and Botanical Characterisation of the Mairá-Potato

According to the Global Biodiversity Information Facility (GBIF) [34], the taxonomy of the Mairá-potato follows the classification described in Table 2.

Botanical characterization of the Mairá-potato was described by Howard [18] (Table 3).

**Figure 1:**
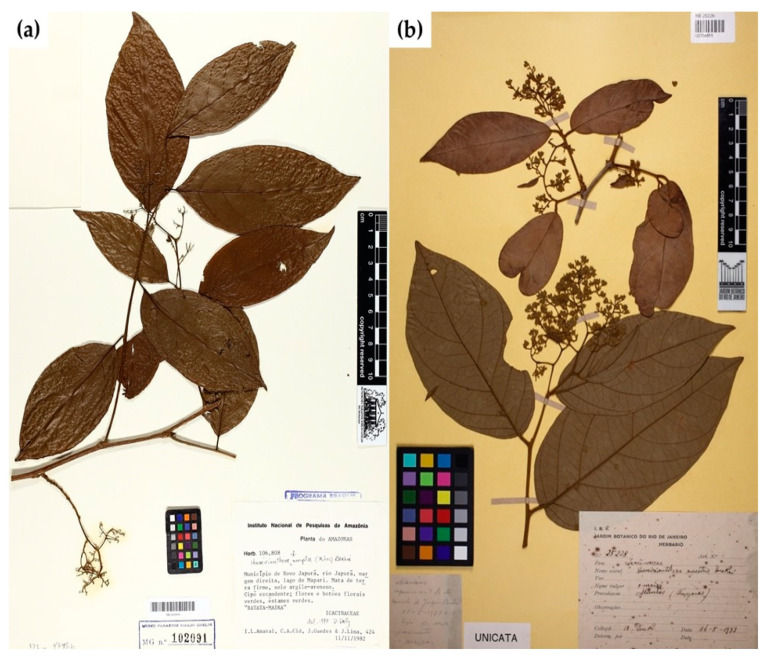
Botanical recognition of *Humirianthera ampla* (**a**) and *Humirianthera rupestris* (**b**). **Source:** [36,37].

Botanical recognition of *Humirianthera ampla* (Figure 1a) and *Humirianthera rupestris* (Figure 1b). 

### 2.3. Food Use of the Mairá-Potato

The Mairá-potato was collected and identified by the British naturalist Richard Spruce during his trip to the Amazon in 1849. He found it among the Tapuyas indigenous peoples who lived on the Janauari River on the lower Negro River. According to Spruce (1851), they knew it by the name *maniaca-açu* (“big cassava”) and used it in the same way as cassava, obtaining flour and tapioca from it. The botanist also noted that the plant was first used by the Purupuru indigenous peoples who lived on the Purus River [38]. 

The fruits, roots, and tubers were used to produce gum, which, according to the Arawa language, was used as *biha* (or *bija*), which designates tuberous plants, roots, and tubers translated as wild tubers of terra firme corroborating with the information provided by the botanist Richard Spruce regarding *Casimirella* rupestris being compatible with information regarding the bija of the Suruwaha. The Mairá-potato occurs in sloping forests and capoeira on sandy and clay soils. In dry land environments, this plant produces a sizeable tuberous plant that can reach a mass of more than 200 kg, from which native peoples extract the starch for the production of *grolado* and *beiju* [12].

Tubers are considered significant food sources for Amazonian gathering groups that also practice the cultivation of some plant species. In 1948, the ethnologist Paul Ehrenreich observed that an indigenous group called Paumari from the Purus basin exploited a large native tuber—the Mairá-potato (*Casimirella rupestris*)—from which they extracted the starch for the production of flour [39]. Historically, travelers recorded the presence of the Mairá-potato in several Amazonian rivers used by indigenous groups to produce gum [12].

The plant species of this genus have starchy tubers and are edible (after successive washing with water as they contain toxic substances) [40]. Rizzini and Mors [11] stated that *C. ampla* and *C. rupestris* are starchy sources used for food, that these plant species are popularly known as the Mairá-potato, and that they can reach weights over 100 kg.

In the work of Leitão-Barboza et al. [41], it was reported that the Katukina indigenous peoples still have several specimens of *Casimirella* sp. in their gardens. However, despite having consumed this tuber for a long time, they currently opt for cassava flour; the Indians consider cassava more productive and it requires less work to process.

Ribeiro [8] carried out an ethnobotanical study of *Casimirella rupestris*. Firstly, he surveyed the indigenous peoples of the Paumari, Apurinã, Jamamadi, and Jarawara ethnic groups located in the region of the middle Purus/Amazonas/Brazil. All the ethnic groups reported the use of the tuber as food, with a similar technique for extracting the starch contained in the tuber with rudimentary techniques. They grated it on thorny roots and squeezed in *tipiti*, an instrument formed by vegetable fibers in which all the material is compressed and the liquid residue is expelled. The study showed that the Paumari indigenous peoples have several specimens of *Casimirella ampla* in their lands. However, several of the indigenous people who lived there reported that they did not know the species. 

Ribeiro [8] evaluated various parameters of interest for food use such as, starch yield ranging from 5.7 to 15.7%; starch yield around 84.31%; amylose content 38.17%; starch morphology, which showed predominant granules in circular, oval, and ellipsoid shapes with an average diameter of 24.48 μm; swelling power at temperatures of 65, 75, 85, and 95 °C was low, being 3.13, 17.71, 21.58, and 24.67 g of water/g of starch, respectively; solubility was low at the same temperatures, being 2.77, 14.53, 14.85 and 16.36%, respectively; paste properties (peak viscosity 256 RVU-rapid viscosity analyzer unit, breakdown 125 RVU, final viscosity 207 RVU, retrogradation tendency 76 RVU, paste temperature 76.4, and peak time 7.4 min); thermal properties (initial temperature 65.51 °C, peak 69.99 °C, and conclusion 75.93 °C; enthalpy variation 17.13 J/g; temperature variation 10.42 °C. In the toxicity test, it was possible to detect toxicity in the samples that went through up to five washes; after the sixth wash of the extracts, toxicity was not detected. This study evidenced the high concentration of starch and amylose, demonstrating this tuber’s technological and functional potentiality with respect to toxicity studies.

In work by dos Santos et al. [42], the diagnosis of starch grains from bread found in the southern region of the Amazonas state was performed. In the publication, the authors surveyed several findings of “Indian bread” (as they called the material) and it was possible to identify the presence of the Mairá-potato. Traces of mechanical impact related to *Casimirella* sp. grains were observed, suggesting its processing in grinders or graters. However, due to such a high quantity of transitory starches, the authors inferred that they could be from various plant parts, such as the leaves. For example, Watling et al. [43] stated that there were several records of bread being wrapped in leaves of different species before being buried; still, dos Santos et al. [42] indicated this process.

### 2.4. Pharmacological Properties of the Mairá-Potato

Empirically, Amazonian communities usually use *Casimirella ampla* extract as an anti-ophidic and even administer therapeutic preparations of this tuber orally [20]. Some authors have claimed that plants of the genus *Casimirella* possess chemical compounds that work against snake venom [44] and have an anti-inflammatory action [45,46].

Raw ethanolic extract of *C. ampla* or its isolated constituents were evaluated for possible anti-ophidic activity in the study of Strauch [28]. The experiments showed a concentration dependent inhibition of *Bothrops atrox*, *Bothrops jararaca*, and *Bothrops jararacussu* venoms by *Humirianthera ampla* extract and inhibition of *Bothrops jararacussu* and *Bothrops atrox* by sitosterol and lupeol, respectively. These data suggested that the decrease in myotoxicity of Bothrops venoms is related to the inhibition of phospholipase A2 activity and proteolytic activities of the venom. The study further suggested that some effects in vivo can depend on these enzymatic activities.

Monks et al. [21] conducted studies identifying Brazilian plant species that demonstrated in vitro activity against human tumor cell lines. When they evaluated *C. rupestris*, they observed cytotoxic activity in one or both cell lines tested. Cytotoxicity was observed when the reduction in SRB absorbance was <10% for HT29 and <5% for NCI-H460 from the control. These values equaled the zero-control absorbance time for each cell line, i.e., the initial cell number. 

Lupeol is a pentacyclic triterpene that occurs in many medicinal plants and is found in many fruits and vegetables. This naturally occurring triterpene is effective in inflammatory responses and has immuno-modulatory properties [47,48]. Triterpenes inhibit tumor growth and cell cycle progression and induce apoptosis of tumor cells in in vitro and in vivo tests, as well as presenting anti-inflammatory, anti-oxidant, and anti-angiogenic effects [47,49]. This compound showed anti-inflammatory properties [50,51,52] and anti-cancer activity against different melanomas (G361, 451Lu, and WM35), T-lymphoblastic leukemia (T-lymphoblastic leukemia), breast carcinomas (MCF-7 and MDA-MB-231), lung carcinoma (A-549), multiple myeloma (RPMI 8226), and cervical carcinoma (HeLa) cell lines [51,53,54]. Some natural esters of lupeol also show promising biological effects, such as anti-malarials [55].

Boakye et al. [56] showed that lupeol has been shown to inhibit NF-κB and increase FGF-2, TGF-β1, and collagen III levels, followed by negative regulation of IL-6 and subsequent positive regulation of IL-10 levels in a wound healing model in diabetic patients. Saha et al. [57] confirmed the anti-inflammatory activity of lupeol against 7KC in M (IFN-γ/LPS) macrophages by suppression of the inflammasome and activation of autophagy, suggesting further studies of lupeol regarding therapeutic strategies for plaque regression and further detailing of the therapeutic anti-inflammatory effect for future human applications. However, they reinforced previous findings on the immuno-modulatory system effects of lupeol on innate immune cells. They referred to the utility of this triterpene as an adjuvant drug to counteract the proatherogenic signaling of oxysterol within atherosclerotic plaque by activating autophagy and inhibiting pro-inflammatory cytokines.

Sharma et al. [58] stated that lupeol and some analogs were shown to possess several popular and recognized biological activities and, in addition, possessed the potential to be consumed as a nutritional supplement to prevent cancer, inflammation, and coronary and liver diseases. This compound also exhibited low cytotoxicity in healthy cells. It acted synergistically when used in combination therapies, enabling it to be applied alone or as an adjuvant to clinically used anti-neoplastic and anti-inflammatory drugs.

Marques et al. [29] studied another compound found in *Casimirella* sp., annonalide (1) and its derivatives (2–10); as to the cytotoxic activity against human tumor cell lines, cell lines representing the most prevalent types of cancer worldwide were used. Cells tested were HL-60 (human leukemia), PC-3 (prostate carcinoma), HepG2 (hepatocellular carcinoma), SF-295 (glioblastoma), and HCT-116 (human colon). Furthermore, HCT-116, HepG2, and PC-3 lines were cells resistant to conventional therapies [59,60]. 

The cytotoxic activity of all compounds was investigated against five tumor cell lines and normal cells. Annonalide and derivatives were the most active compounds when tested against the leukemic cell line HL-60. Nine annonalide derivatives were prepared by the semi-synthesis method. Most compounds showed IC50 of 4.0 μM or less, suggesting their potential as anti-tumor agents. The chemical modifications in the natural product were mainly done on the side chain linked at C-13. The interaction was verified and it can be inferred that annonalide interacts with ctDNA by intercalation. The cytotoxic activity of annonalide against human tumor cell lines may be associated with its interaction with DNA [29].

Jiménez-Escrig et al. [61] established the calculation of the index of dietary sterol status as the ratio of plant sterol/cholesterol, i.e., ((β-sitosterol + stigmasterol)/cholesterol). They evaluated four groups of people (composed of Seventh-Day Adventists; vegetarians; ovo-lacto-vegetarians, and the general population). A ratio ranging from 0.49 to 16 was observed, which the authors attributed to different individual diets. Regarding β-sitosterol, [62] stated that the daily intake of β-sitosterol was about 79.7% of the total phytosterol per person. Furthermore, they stated that β-sitosterol exhibited a protective influence on experimentally induced colon cancer. Moreover, it also possessed anti-cancer, anti-atherosclerosis, anti-inflammatory, and anti-oxidant properties.

Gupta et al. [63] administered β-sitosterol 10, 15, and 20 mg/kg for 21 days to streptozotocin-induced diabetic rats; all doses administered decreased serum glucose, nitric oxide, and glycated hemoglobin and increased serum insulin and pancreatic anti-oxidant levels, with a significant decrease in thiobarbituric acid reactive substances, demonstrating that β-sitosterol is a promising anti-diabetic and anti-oxidant agent. 

Shi et al. [64] noted that incorporating β-sitosterol into mitochondria increased mitochondrial inner membrane fluidity without affecting mitochondrial outer membrane fluidity and consequently increased mitochondrial membrane potential (ΔΨm) and mitochondrial ATP content. This effect may be beneficial for neurodegenerative diseases such as Alzheimer’s disease. Ayaz et al. [65] also studied the effect of β-sitosterol on neuro-degenerative disorders; they observed strong anti-cholinesterase and anti-oxidant potential and double efficiency (inhibition of enzymes and elimination of free radical capacity) and concluded that the use of β-sitosterol can improve cognitive deficits, short-term memory, and locomotor deficits. 

Zhao et al. [66] mentioned that natural supplies of *Casimirella* sp. molecules and derivatives can be further investigated because, unlike other compounds that are formed from stress-induced metabolites with limited amounts such as momilactone, *Casimirella rupestris* presents high structural diversity and high content, providing opportunities for the discovery of promising compounds, as well as precursors for the semi-synthesis method. In addition, it has the potential for developing herbicidal crops and anti-fungal, anti-bacterial, and anti-tumor agents.

#### Anti-Parasitic and Anti-Fungal Potential of Mairá-Potato Compounds

The study by Ramos et al. [67] evaluated the *in vitro* leishmanicidal activity of extracts and substances isolated from *Casimirella ampla.* It was observed that the ethanolic extract presented results against *Leishmania amazonenses* and *Leishmania braziliensis*, the concentration of 50 mg/mL promoted the death of 97.2% of *L. braziliensis* parasites after 48 h of treatment and 99.2% of inhibition for *L. amazonenses* parasites.

Gonçalves et al. [68] also studied the in vitro leishmanicidal activity of the extract of *C. ampla* roots. It was observed that the ethanolic extract at a concentration of 100 µg/mL completely inhibited the growth of *Leishmania braziliensis* parasites, showing the possibility of application of compounds present in *C. ampla* as an anti-leishmanial drug.

### 2.5. Phytochemicals Present in Parts of the Mairá-Potato Plant

Some scientists have studied the chemical compounds in the plant species C. *ampla* and *C. rupestris*. In the study by Zoghbi et al. [13], *C. rupestris* tubers were isolated and identified and several secondary metabolites were classified as degraded γ-lactonic diterpenoids, with structures containing between seventeen and nineteen carbon atoms. Afterward, Zoghbi et al. [14], using tubers and stems of *C. ampla* and *C. rupestris*, isolated sodium and potassium thiocyanate and sodium and potassium nitrite and nitrate and obtained concentrations of 1 g from the tuber and 2.75 g from the stem, respectively.

Varejão et al. [16] studied leaves, stems, and tubers of young and adult *C. ampla* and *C. rupestris* in the rainy and dry seasons. They observed that the concentration of minerals in the organs analyzed for both species obeyed the relation N > Ca > Mg > P > K. In the tuber, for example, this ratio was 1.55 > 0.73 > 0.14 > 0.13 > 0.06% and 1.96 > 0.43 > 0.18 > 0.13 > 0.03% in the rainy season for adult and young *C. ampla*, respectively. The ratio for *C. rupestris* was different in the rainy season, being 0.65 < 0.70 > 0.48 > 0.18, 0.18% and 1.20 > 0.74 > 0.64 > 0.20 > 0.19% for adult and young, respectively. The behavior was different for both species in the dry season, being 1.50 > 0.11 < 0.25 > 0.14, 0.14% and 1.60 > 0.69 > 0.10 > 0.08 < 0.14 for adult and young *C. ampla,* respectively, and different for *C. rupestris* in the dry season, being 0.53 > 0.34 > 0.10 > 0.04 < 0.06% and 1.64 > 0.01 < 0.38 > 0.03 < 0.12%, for adult and young, respectively.

The average content of these elements indicated no significant variability dependent on seasonality or physiological age. A study by Varejão et al. [17] evaluated the same organs of the same species, physiological ages, and seasons. They observed that sulfate (SO^4−^) uptake by *C. ampla* and *C. rupestris* individually did not depend on seasonality or physiological age. They also noted that sulfur (S) content followed the different relationship between species in the rainy season, regardless of physiological age.

Graebner et al. [19], from the crude ethanolic extract obtained from the root of *C. ampla*, isolated the following substances: lupeol, β-sitosterol, glycosylated sitosterol, and the already known but not described in previous studies of this species, diterpene annonalide, as well as two new ones: acrenol and humirianthol. Later, ref. [20] isolated a phthalate, lupeol, β-sitosterol, glycosylated sitosterol, and three diterpenes (annonalide, humirianthol, and acrenol) from the ethanolic extract of *C. ampla*. Graebner [24] studied tubers of the *C. ampla* species and isolated secondary metabolites of β-amyrin, glycosylated sitosterol, and a new component, 1 β-O-β-D-glycopyranosylplumeric. 

Based on the methodology used by Graebner et al. [19], in which humirianthol and acrenol were isolated and purified from *C. ampla*, Burrow et al. [22,23] were able to synthesize the compound acetylated humirianthol and subsequently synthesized diacetylated acrenol. In addition, the identification of the compound icacinol was reported, which had not been described in the works surveyed previously for this species.

Adou et al. [25], from ethyl acetate and methanol extracts of *C. ampla*, isolated the following compounds: humirianthol, annonalide, acrenol, the oxidized compound of annonalide, and icacinol, as well as five other compounds, to which they assigned the nomenclatures humirianthone, 1 hydroxy-humirianthone, 15R-humirianthol 15 R—humirianthol, patogonol, and patogonal. The tubers of C. ampla species were also studied by Marques [26], who isolated and identified mixtures of the steroids β-sitosterol and stigmasterol, annonalide, lupeol, and 3- β-O-β-D-glucopyranose sitosterol. Ribeiro [8] evaluated the mineral content in *C. rupestris* starch. In addition to other minerals, the author observed contents of 90, 20.9, and 4.1 mg/100 g of calcium (Ca), iron (Fe), and zinc (Zn), respectively. However, according to him, the bioavailability of minerals should be evaluated because little is known about this PANC.

Table 4 shows the structural formulas of phytochemical compounds isolated and identified from parts of the Mairá-potato plant.

#### Toxicity of Mairá-Potato and Indications for Evaluation to Ensure Application as a New Product or Ingredient

There is a scarcity of scientific data systematically compiled on the Mairá-potato, either *C. ampla* or *C. rupestris*. Although it has been used as food, little is known about the effect of the consumption of this PANC in the case of habitual consumption, so it is necessary to cite studies on toxicity since the safety of the material for human consumption needs to be ensured regarding the applications that are suggested in this work. This scarcity may be closely related to the little known level of toxicity in this tuber;, the most recent work of 2018 was the dissertation by Ribeiro [8], who sought to address the issue of toxicity and how to minimize it in the Mairá-potato.

Spruce [38] reported that the Mairá-potato is a food species that needs treatment to remove flour and starch toxicity, corroborating the study of Ribeiro [8] regarding the presence of toxicity in this species. According to him, after successive washings of the starch and the bark, the material showed no toxicity after the fifth wash. In the study by Zoghbi et al. [13], the toxicity of this species was mentioned regarding the tubers of *C. rupestris*. The authors stated that, in a study from Uabatuba, which was not published in the consulted databases, such toxicity has yet to be wholly clarified.

However, according to Paschoal and Souza [10], it is essential to search for knowledge regarding the possible presence of toxic phytochemicals and anti-nutritional factors in the case of inappropriate consumption of non-conventional food plants. Considering that the Mairá-potato has already been used for food, as mentioned during the work, and that some authors have cited the presence of toxicity, it is suggested that safety tests should be carried out for pharmaceutical and food purposes.

Huggett and Verschuren [69] stated that, when it comes to the launch of a novel food or ingredient, a safety evaluation is necessary, including information on name, origin, source, production or preparation methods, previous history, specification (water content, nitrogen, lipid, carbohydrate, and ash composition), the purpose as well as its uses, and toxicological studies for its application, such as toxicokinetic, genotoxicity, and allergenic potential. Several guidelines for toxicological assessments are described by the Organization for Economic Co-operation and Development (OECD) [70].

### 2.6. What Advances Can Contribute to the Development of New Technologies on the Basis of What Has Been Learned?

The biomolecules present in vegetables are essential for the technological advancements in the food and pharmaceutical areas because they allow the discovery of new products and ingredients, as much for food consumption as for medicine production. However, even though many activities of plants have already been tested empirically by native communities, it is not enough to prove their safety, as already mentioned. This way, by respecting the safety evaluations, several suggestions will be presented for the application of the Mairá-potato for the use of some of the compounds already identified and isolated from this tuber, as well as the use of starch for food use, polymeric matrix, and encapsulation of natural actives, other than use in the 3D printing of food.

Developing a relevant product, be it a drug, vaccine, or even a biomaterial, is a complex process that requires financial and human resources. From the beginning of the idea to the final stage, these products demand considerable time, high cost, and strict process control. Although the development of new technologies can be expensive, the final product will undoubtedly contribute to humanity’s scientific and social advancement [71]. 

From now on, we will look at possible applications for the Mairá-potato. The lessons learned will be used to discuss more clearly the possible applications of this tuber and to propose ways to contribute to developing new technologies.

#### 2.6.1. Application of Starch for the Development of Biodegradable Films

The environmental concern of consumers regarding plastic disposal has produced an interest in biodegradable films in the industrial environment, since such films make it possible to control factors such as oxygen, humidity, and carbonic gas associated with the possible incorporation of anti-microbial and functional bio-compounds into the polymeric matrix [72,73]. This matrix can be lipidic, proteinic, or polysaccharide, such as starch. Starch is widely used because it has several characteristics such as film-forming capacity, neutral sensorial aspects, low cost, and abundance [74,75,76]. 

In addition to its importance in the food industry, starch has wide applications in the paper, chemical, textile, pharmaceutical, and biotechnological industries, making it a very versatile raw material [77]. The type of starch for specific industrial purposes is selected based on its availability and physicochemical properties, which are influenced by the source from which the starch is extracted [78,79]. In addition, there is immediate availability in nature, biodegradability, renewable character, and possibilities of modifications due to the abundance of chemical -OH [80]. 

Polysaccharides are employed as ice cream stabilizers, food emulsions, micro-encapsulation of flavors and dyes, clarifiers, and beverage stabilizers. Therefore, information about the molecular structure, thermal stability, interaction with water, and rheological behavior is essential knowledge to prospect and develop applications for each type of polysaccharide, either isolated or in mixtures. Another point to consider is the constant search for new sources of polysaccharides that may have similar or better effects than those already known. This is important because it also shows regional appreciation, source of income, and new business opportunities [81].

Studies on the technological potential of starch from alternative sources can provide an alternative to the traditional primary sources of starch feedstock [82]. Unlike conventional starches that are widely produced around the world, new botanical sources are generally consumed as a staple food by local populations and indigenous cultures in rural areas of emerging countries, where diets are rich in vegetables and family farming is common [83,84,85].

Knowing starch’s functionalities is paramount to determining its possible use. Amylose is an essential characteristic in terms of optimum film-forming properties; the shape and size of granules are also relevant and all these properties depend on the botanical origin [49,86]. The Mairá-potato emerges as a non-conventional source of starch, which can be significantly explored from the point of view of the functionalities sought for a starch-based polymeric matrix, because, as mentioned in the study of Ribeiro [8], this tuber has several properties.

The Mairá-potato starch is a promising polymer for the development of films. Liporacci et al. [87] attributed this capacity to the starch with high amylose content studied by them (*Dioscorea alata* starch), with values of 37.46 g/100 g, a value near to that found by Ribeiro [8], which was 38.17% amylose. The lower the number of other constituents and the higher the amount of amylose, the better the film and coating formation because amylose is directly linked to the chemical and physical characteristics of the film [88,89,90]. 

Other essential features for film formation are paste properties and thermal properties. According to Ribeiro [8], the initial paste temperature (76.4 °C) presented by the Mairá-potato is associated with internal inter-molecular forces of starch granules, which is closely linked to the amylose. The paste formed by starch from this tuber showed mechanical and thermal stability; it took 7.39 min to reach the peak of viscosity (256 RVU) and showed a low value of breakdown (125 RVU) and a low tendency to retrogradation (76 RVU). As for the gelatinization temperature, it was between 69.99 and 75.93 °C, which favored application in processes that demand high temperatures, as happens in the formation of biodegradable films of cassava starch between 75 and 95 °C [91] and potato starch at 80 °C [92].

Film-forming material can be applied in encapsulation and grouped according to particle size, micro-encapsulation, and nano-encapsulation. Micro-emulsions have a diameter between 3 and 800 μm, while nano-emulsions have a particle size ranging from 10 to 1000 nm (1 μm) [93]. Ribeiro [8], in the study with the Mairá-potato, observed a starch grain size of 24.48 μm, i.e., the starch grains of Mairá-potato are within the classification of micro-emulsions. Micro-emulsions are indicated for use in the encapsulation of veterinary drugs, taken as an efficient approach to delivery. In other terms, it is a more efficient way for the active compound to be released in a controlled manner, in the veterinary case, to prevent animals from being affected with another substance other than the bioactive substance of interest [94]. In addition, micro-encapsulation technology can be applied to regenerative medicine, in which hydrogels are produced to encapsulate stem cells [95]. 

#### 2.6.2. Application of Bioactive Ingredients Contained in the Mairá-Potato

Throughout history, bioactive compounds have been used for treatment because of their therapeutic effects. The new trend is to recover bioactive compounds that have physiological effects on living organisms [96]. Bioactive compounds can be recovered from food processing by-products, medicinal plants, and other natural resources [97]. 

The quality and yield of bioactive compounds depend on two crucial factors, the extraction method and the extraction parameters, including the plan matrix type, the solvent used, time, and temperature [98]. The initial steps for using active compounds from plant matrices are extraction followed by pharmacological testing, isolation, characterization, and clinical evaluation. It should be kept in mind that choosing the delivery system of a bioactive compound depends on some factors such as solubility, stability of the bioactive compound, and its product applications [99].

Considering the proposal to use the encapsulation technique, a compound such as lupeol has been discussed by several authors [57,58]. Other encapsulated compounds discussed were annonalide [29] and β-sitosterol [64,65]. The latter is already known to be used in the production of pharmaceuticals. The proposal is to apply these activities in the Mairá-potato in possible products. We then suggest encapsulation.

Encapsulation is a method in which a component is surrounded by another material and produces particles with diameters of nano-, micro-, or millimeter sizes. The encapsulated components can be in different forms, such as an active agent, base material, filler, internal phase, or payload phase [99]. The nutraceutical and pharmaceutical sectors use active extracts to develop functional foods and herbal medicines, which have the potential to heal and provide health benefits [98].

Encapsulation technology can be grouped into two broad groups. The first is proper encapsulation, in which a liquid or solid core is trapped in a gelatinous capsule. The other includes contemporary techniques, in which active ingredients are trapped in the encapsulating matrix or wall material made of various carriers [100]. The success of an effective encapsulation process depends on the selection of three target bioactive factors: molecules, wall materials, and a suitable encapsulation method [101].

Encapsulation technology has been prominent for decades for solving the limitations encountered in delivering active pharmaceutical ingredients (APIs), food ingredients, and cosmetics and veterinary, hygiene, and cleaning products. Among the technologies most commonly used in producing delivery systems for APIs are coacervation, solvent evaporation, solvent emulsion, ionic gelation, extrusion, high-pressure homogenization, spray drying, and spray cooling or spray cooling/spray freezing [102].

Therefore, the active molecules of the Mairá-potato have potential for various applications, whether pharmaceutical, cosmetic, or food, because there is a range of ways to encapsulate bio-compounds, being necessary to deepen the knowledge specifically about the target molecule.

#### 2.6.3. Material for Printing 3D

Three-dimensional (3D) printing, or additive manufacturing, uses digital data to create a three-dimensional physical object, usually by laying down layers of material in succession. Three-dimensional printing is a unique technique that allows users to create highly complex materials that are difficult to create otherwise using traditional mechanical manufacturing techniques. In addition, 3D-printed objects are immensely customizable through design parameters and different types of printing materials [103]. Three-dimensional printing has already been adopted in several areas, such as the military [104], medicine, pharmaceuticals and biomedicine [105], chemistry [106], and food [107,108].

Three-dimensional printing of food, or additive manufacturing of food, has long been touted as a method for on-demand production and complex customization of food products, with the ability to produce products with unique shapes, combinations of food types, customized flavors, textures, and nutrition. Three-dimensional printing offers the potential for localized on-demand production and is proposed to create personalized nutritional profiles of meals [109]. 

Depending on their design, 3D printers can print various materials, metals, and plastics [110]. Three-dimensional printing techniques include extrusion-based printing, selective laser sintering, and binder jet and inkjet printing. Of these, extrusion-based printing is the most commonly used method for 3D food printing, which involves a liquid or semi-solid material being extruded through a nozzle, moving in the x-, y- and z- directions, to build a food product layer by layer, usually followed by post-printing processing such as baking or frying [111].

Formulating food inks with the required physicochemical and functional properties is one of the most critical factors in any successful 3D food printing application [111]. Food inks, which may contain one or more ingredients, are typically added to an extruder, pushed out applying an external force, and then printed into pre-designed shapes using spatial data stored in the instrument’s software [112]. The force required to extrude food ink through a nozzle is typically provided by connecting the printer to an air pressure unit. 

The researchers identified several important parameters affecting the success of 3D food printing, including hardness, elasticity, plasticity, and visco-elasticity [112,113,114,115]. To successfully print food, essential material properties such as rheology, surface tension, and phase behavior of the food ink need to be considered. In addition, the operating parameters of the 3D printer used, such as nozzle height, nozzle diameter, flow rate, and printing speed, are also crucial as they affect the structure and stability of the printed food.

Ensuring the extrusion capability of food materials is essential for 3D printing capabilities, such as shape fidelity and retention over time [116,117]. Three-dimensional structures require precise extrusion of individual line filaments (1D structures), also known as extrudability. Extrudability is assessed by extrusion and filament uniformity [118]. 

Liu et al. [119] investigated the impact of rheological properties of 3D-printed mashed potatoes with the addition of different concentrations of potato starch and concluded that the most desirable materials for 3D food printing should not only possess adequate yield strength and modulus of elasticity to be able to hold the printed shapes, but also should have low consistency index and flow behavior index to be easily extruded out of the nozzle onto a printer-type extrusion base. They observed that the mashed potato, with 2% potato starch added, showed excellent extrudability and printability.

Liu et al. [120] suggested that yield stress and pseudo plasticity were closely related to the extrudability of food hydro-colloids. Furthermore, printing parameters, including printing pressure, nozzle speed, and nozzle height, were identified as factors that co-determined the extrusion capacity and printability of alginate/gelatin hydrogels [116]. 

Although the paste-forming ability of Mairá-potato starch has been tested by Ribeiro [8], its flowing and pseudo-plasticity behavior is not known, nor is it known whether the printing nozzle can extrude it. However, it is suggested to use this starch as an emulsifying ingredient or even to add texture to 3D-printed products for food (subject to safety and adaptability tests).

## 3. Materials and Methods

This review followed the statement of preferred reporting items for systematic re-views and meta-analyses (PRISMA) [121]. On 9 June 2021, information on the *Casimirella* sp. species (botanical, food, pharmacological, and phytochemical aspects and isolates) were searched in the Google Academic, Scielo, Science Direct, Scopus, PubMed, and Web of Science databases. After searching the databases with the terms *Casimirella rupestris* and *Casimirella ampla,* only one article was found that related to the subject, thus it was decided to search for the terms *Humirianthera rupestris* and *Humirianthera ampla*. No restriction was applied. In other words, publications in any language that were published until 9 June 2021 (the date of the research) in the databases mentioned above that were related to botanical, food, pharmacological, and phytochemical aspects were included.

Table 5 shows the descriptor terms and Boolean operators used for the search in the Google Academic, Scielo, Science Direct, Scopus, PubMed, and Web of Science databases.

Duplicate articles were excluded and, after reading the title and abstract, articles that did not meet the inclusion criteria were excluded. In addition to the selected articles, theses and dissertations that addressed the searched terms were included. 

**Figure 2:**
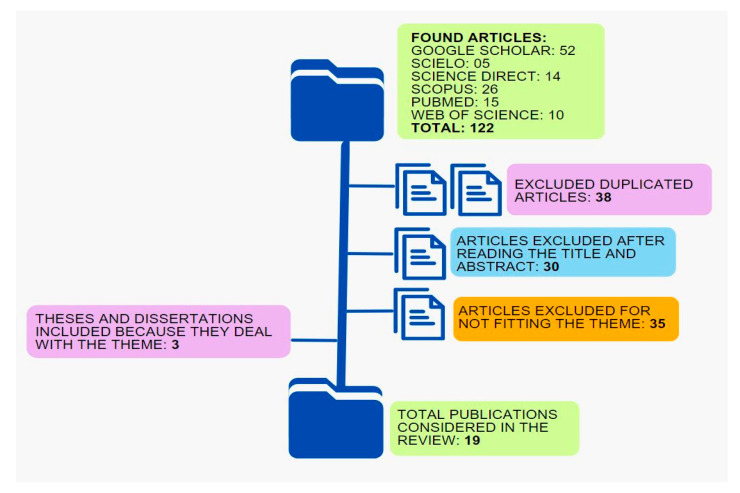
Flowchart of the evaluation of the articles resulting from the search in the Google Academic, Scielo, Science Direct, Scopus, and Web of Science databases.

Figure 2 shows the flowchart of the evaluation of the articles resulting from the bibliographic survey.

## 4. Conclusions

The Mairá-potato (*Casimirella* sp.) appears as a species that, despite being cited by some authors as edible by indigenous peoples, has been little studied for food purposes. However, knowledge of its medicinal use is also an excellent research alternative on the population’s use in food and its possible application in products from various areas such as pharmaceuticals, cosmetics, and food. 

This review synthesized knowledge about the Mairá-potato, which can contribute to the direction of new research on the technological applications suggested as a polymeric material, biomaterial encapsulation, and bioactive and 3D printing. As such, this work gathered information about this PANC that shows great potential for use in various areas of study. 

Several characteristics of the Mairá-potato have not yet been studied. There is a wide field of research on possible enzymes, extraction, and isolation of other food products, such as bread, that are produced technologically, application as an emulsifier or stabilizer, in the formulation of breaded products, creams, soups, and several other uses, where conventional starches are applied and the Mairá-potato can be inserted.

## Figures and Tables

**Table 1:** Systematization of the 19 papers on food, botanical, pharmacological, and phytochemical aspects of *Casimirella* sp. resulting from searches in the Google Academic, Scielo, Science Direct, Scopus, PubMed, and Web of Science databases.

No	Title	Principal Findings	Publication	Ref.
01	Humirianthenolides, new degraded diterpenoids from *Humirianthera rupestris*	The tuber of *Humirianthera rupestris* (Icacinaceae) contains the degraded diterpenoids 3fi,20-epoxy-3uhydroxy-14-oxo-9βpodocarpan-19,6β-olide (humirianthenolide A), 3β,20-epoxy-3α,14u-dihydroxy-9β-podocarpan19,6β-olide (humirianthenolide B), 3β,20; 16,14-diepoxy-3cl-hydroxy-17-nor-15-oxo-9β-abiet-l3-en-l9,6β-olide (humirianthenolide C), 3β,20-epoxy-3a,14-dihydroxy-13-oxo-9β-podocarp-8(14)-en-l9,6β-olide (humirianthenolide D), 3β,20-epoxy-3a-hidroxy-14-oxo-8α,9β-podocarpan-l9,6β-olide (humirianthenolide E), and 3β,20-epoxy-3α,14β-dihydroxy-8α,9 β podocarpan-19,6β-olide (humirianthenolide F). ‘H NMR and ‘3C NMR spectroscopy were effective for the determination of the humirianthenolide structures.	*Phytochemistry*	[13]
02	Chemical study of *Humirianthera* ampla. (Miers) Baehni (Icacinaceae)	From the tubers of *H. ampla* (Miers) Baehni (Icacinaceae), the humiriantenolides A, C, and D were isolated in addition to sitosterol.	*Acta Amazonica*	[14]
03	The presence of toxic inorganic substances in the genus *Humirianthera* (Icacinaceae)	The isolation of thiocyanate crystals, sodium and potassium nitrate, and nitrite in the tuber and stem of *Humirianthera ampla* has been reported. The nitrate and nitrite contents in the leaves of *H. ampla* and *H. rupestris* at young and adult ages were also determined. The entire contents of N-NO_3_ observed for *H. ampla* and *H. rupestris* were 8.83 mg % and 6.42 mg %.	*Acta Amazonica*	[15]
04	Seasonal variation of macro- and microelements in the genus *Humirianthera* (Icacinaceae) as a function of age	Twenty-six specimens of *H. ampla* and nine of *H. rupestris* at young and adult ages collected during the dry and rainy seasons were analyzed for the total content of N, F, K, Ca, Mg, Mo, Cu, Zn, Mn, Fe, B, Al, Co, Cr, Na, Pb, Si, and Sr in the different vegetative parts of the plants. The concentration of macro-elements in the leaves, stems, and tubers of *H. ampla* and *H. rupestris* obeys the relationship N > Ca > Mg > F > K. The average concentration of these elements indicates no significant variability in the organs analyzed nor in relation to the ages, except for N, which presents a higher content in the leaves and in relation to the other elements. The micro-elements are uniformly distributed in the various organs, with Fe, Al, and Na having the highest concentration.	*Acta Amazonica*	[16]
05	Seasonal variation of sulfate, total sulfur and organic matter in the genus *Humirianthera* as a function of age	Twenty-eight (28) *H. ampla* and nine (09) *H. rupestris* adult and young specimens were collected to determine the levels of S04β and total-S in the leaf, stem, tuber, and soil where they grew. In *H. ampla*, the level of S04β varied from 0.22 to 0.78% and in *H. rupestris* from 0.22 to 138%. The level of S in *H. ampla* varied from 0.74 to 0.96% and in *H. rupestris* from 0.75 to 1.02%. The level of S04β IH *H. ampla* followed the patterned leaf > tuber > stem independent of time of year and physiological age, while in *H. rupestris*, the pattern was tuber > leaf > stem. The 5 showed a different behavior, maintaining the pattern tuber > stem > leaf for *H. ampla* and tuber > leaf > stem for *H. rupestris*.	*Acta Amazonica*	[17]
06	A revision of *Casimirella*, including *Humirianthera* (Icacinaceae)	*Casimirella Hassler* (1913) is accepted and *Humirianthera Huber* (1914) is considered a synonym. *Casimirella diversifolia* and *C. lanata* from Brazil are described as new species. *Casimirella ampla* (Miers) based on *Leretia ampla Miers*, *C. crispula* (Howard), based on *Humirianthera crispula* Howard, and *C. rupestris* (Ducke), based on *Humirianthera rupestris* Ducke, are new combinations.	*Brittonia*	[18]
07	Diterpenoids from *Humirianthera ampla*	Two diterpenoids (humirianthol and acrenol) and the known annonalide were isolated from *Humirianthera ampla*. Humirianthol and acrenol were determined by 1D and 2D NMR spectroscopic techniques to br: 3 beta,20:14 beta,16-diepoxy-3 alpha,15 alpha-dihydroxy-7-pimaren-19,6 beta-olide, and 3 beta,20-epoxy-3 alpha,15,16-trihydroxy-7-pimaren-19,6 beta-olide, respectively.	*Phytochemistry*	[19]
08	Diterpenes isolated from *Humirianthera ampla*. Miers	From *Humirianthera ampla*, Icacinaceae have isolated a phthalate, lupeol, ß-sitosterol, glycosyl–sitosterol, one known annonalide diterpene, and two new diterpenes named humirianthol and acrenol. Humirianthol and acrenol were determined by 1D and 2D NMR spectroscopic techniques to be 3 ß, 20:14 ß, 16-diepoxy-3 a, 15 a-dihydroxy-7-primary-19, 6 ß-olide, and 3 ß, 20-epoxy-3 a, 15, 16-trihydroxy-7-primary-19, 6 ß-olide, respectively. Acrenol has anti-microbial activity.	*Revista Brasileira de Farmacognosia*	[20]
09	Antitumour screening of Brazilian plants	Organic and aqueous extracts of 145 Brazilian plants (538) from 34 families were evaluated for antitumor activity against the human tumor cell lines HT29 and NCIH460. Of the extracts tested, 117 (22%) demonstrated cytotoxicity against one or both cell lines at a 100 mg/mL concentration. These results also confirm the continuing importance of natural product screening models, alongside targeted drug development, in discovering new anti-neoplastic pharmacophores.	*Pharmaceutical Biology*	[21]
10	The acetyl derivative of humirianthol	The title compound, 15alpha-acetate-3beta,20:14beta,16-diepoxy-3alpha-hydroxy-9-epi-7-pimaren-19,6beta-olide, C22H28O7, formed from the acetylation of humirianthol, isolated from the tubers of *Humirianthera ampla*, crystallized in the chiral space group P2(1)2(1)2(1). The structure comprised a pimarane ring system with a methylene ether bridge over ring A, a double bond in ring B, and two five-membered furanyl rings, one fused to rings A and B and the other to ring C. The absolute configuration was set using the absolute configuration of C15, as determined by the Horeau method.	*Acta Crystallographica Section E* structure reports online	[22]
11	Absolute configuration of diacetylated acrenol as its chloroform solvate	The title compound, (15S)-15,16-diacetate-3beta,20-epoxy-3beta-hydroxy-9-epi-7-pimaren-19,6beta-olide chloroform solvate, C24H32O8.CHCl3, formed from diacetylated acrenol, isolated from the tubers of *Humirianthera ampla*, crystallized as a chloroform solvate. The structure was based on a pimarane skeleton and was identical to the previously determined structures of icancinol and the acetylated derivative of humirianthol. The anomalous dispersion of the Cl atoms allowed the absolute configuration to be determined.	*Acta Crystallographica Section E* structure reports online	[23]
12	Study of chemical constituents isolated from medicinal plants of the Purus valley region in Acre (Amazonia)	From the tubers of the species *Humirianthera ampla,* the diterpenes humirianthol (3,18: 14,16 diepoxy 3,15-dihydroxy 7-pimarene-17,6β-olide) (18) and acrenol (15,16 diol-3β,20 epoxy-3α-hydroxy 9 epi-7 -pimarene-19,6β-olide) (19) and the constituents β-amyrin (37), glycosylated β-sitosterol (45), glycosylated plumeride (32), and glycoplumeric acid (1β-O-β-Dglycopyranosylplumeric) (33) were isolated and identified.	Thesis	[24]
13	Cytotoxic diterpenoids from two lianas from the Suriname rainforest	Bioassay-guided fractionation of the MeOH and EtOAc fractions of extracts of two lianas collected in Suriname has led to the isolation of five new diterpenoids, humirianthone 1, 1-hydroxy-humirianthone 2, 15R-humirianthol 3, patagonol 4, and patagonal 5, and the five known diterpenoids, humirianthol 7, annonalide 8, acrenol 9, icacinol 10, and the oxidized annonalide 11. All 10 diterpenoids showed cytotoxic activity against the A2780 human ovarian cancer cell line; compounds 1, 3, 8, and 9 also showed activity against phytopathogenic fungi.	*Bioorganical & Medicinal Chemistry*	[25]
14	Phytochemical and Biological Study of *Humirianthera ampla Miers* (Icacinaceae).	The phytochemical investigation of the roots of *Humiranthera ampla* (Icacinaceae) resulted in the isolation and identification of a mixture of beta-sitosterol and stigmasterol, annonalide, lupeol, and the 3-beta-O-beta-D-glucopyranosyl sitosterol. The structures of these compounds were established by spectrometric analysis (IR, MS, NMR 1H, and 13C), including bidimensional NRM techniques (COSY, HMQC, HMBC, and NOESY) and for comparison with data described in the literature. All extracts were tested using the Ellman assay. Only ethyl acetate extracts and their fractions showed acetylcholinesterase inhibition. The ethanolic extract was the most active. The ethyl acetate extract showed xanthine oxidase inhibition.	Dissertation	[26]
15	Anti-nociceptive action of ethanolic extract obtained from roots of *Humirianthera* *ampla Miers*	The anti-nociceptive actions of ethanolic extract (EE) of roots of *Humirianthera ampla* in chemical and thermal models of pain in mice were investigated. Oral treatment with ethanolic extract inhibited, in a dose-dependent manner, glutamate-, capsaicin- and formalin-induced licking. However, it did not prevent nociception caused by radiant heat in the tail jerking test. The ethanolic extract (30 mg/kg) caused marked inhibition of the nociceptive bite response induced by glutamate, (±) -1-aminocyclopentane- trans -1,3-dicarboxylic acid (trans-ACPD), N- methyl- d- aspartate (NMDA), and substance P. The anti-nociception caused by the ethanolic extract was significantly attenuated by naloxone, L- arginine, WAY100635, ondansetron, or ketanserin, but not by caffeine or naloxone methiodide. The ethanolic extract of *Humirianthera ampla* roots produced anti-nociception against neurogenic and inflammatory models of nociception.	*Journal of ethnopharmacology*	[27]
16	Anti-ophidic activity of the extract of the Amazon plant *Humirianthera ampla* and constituents	Although serotherapy against snakebites was discovered more than one hundred years ago, anti-venom is not available all over Brazil. The use of plants from folk medicine is common mainly in the Brazilian Amazon area. One of these plants is named *Humirianthera ampla* (HA).	*Journal of ethnopharmacology*	[28]
17	Annonalide and derivatives: semi-synthesis, cytotoxic activities, and studies on interaction of annonalide with DNA	The cytotoxic activity of the pimarane diterpene annonalide (1) and nine of its semisynthetic derivatives (2–10) was investigated against the human tumor cell lines HL-60 (leukemia), PC-3 (prostate adenocarcinoma), HepG2 (hepatocellular carcinoma), SF-295 (glioblastoma), and HCT-116 (colon cancer) and normal mouse fibroblast (L929) cells. The preparation of 2–10 involved derivatization of the side chain of 1 at C-13. Except for 2, all derivatives were reported for the first time. Most of the tested compounds presented IC50s below 4.0 μM, being considered potential anti-tumor agents. The interaction of annonalide (1) with ctDNA was evaluated using spectroscopic techniques; the formation of a supramolecular complex with the macromolecule was confirmed. Competition assays with fluorescent probes (Hoechst and ethidium bromide) and theoretical studies confirmed that 1 interacted preferentially via DNA intercalation with stoichiometric ratio of 1:1 (1:ctDNA).	*Journal of Photochemistry and Photobiology B: Biology*	[29]
18	Ethnobotanical and physicochemical study of the *Mairá*-potato (*Casimirella* spp.-Icacinaceae)	An ethnobotanical survey and physical–chemical characterization of the *Mairá*-potato was carried out. The form of starch extraction was very similar among the different ethnic groups. Only the *Apurinã* reported the existence of cultivation (vegetative propagation) and management, where the liana was kept alive in the fields. The physical–chemical study indicated that the tuberous root was a source of starch (68.23% on a dry basis). The yield obtained from starch extraction was up to 15.4% and the mineral contents of calcium, copper, iron, manganese, and zinc were higher than those of manioc and potato starch. The toxicity test revealed that from the fifth wash of the starch, the material extracted from the supernatant was non-toxic to *Artemia salina*. The functional properties of the starch revealed granules that possessed stability to thermal and mechanical action and that were relatively large, with an average size of 24.48 μm, and possessed high amylose content (38%).	Dissertation	[8]
19	Flora of the Ducke Reserve, Amazonas, Brazil: Icacinaceae	Three species belonging to two genera were recorded: *Casimirella rupestris*, *Pleurisanthes emarginata*, and *P. parviflora. Casimirella rupestris* is easily differentiated from *Pleurisanthes* species by presenting branches covered by stellate trichomes and paniculate inflorescence (vs. glabrous or puberulent branches in *Pleurisanthes*).	Rodriguésia	[30]

**Table 2:** Taxonomic classification of the Mairá-potato.

Kingdom	Plantae
Phylum	Tracheophyta
Class	Magnoliopsida
Order	Icacinales
Family	Icacinaceae (Benth.) Miers
Genus	*Humirianthera Huber* (synonym *Casimirella Hassler*)
Species	*Humirianthera ampla* (Miers) Baehni (synonym *Casimirella ampla* (Miers) R.A.Howard) and *Humirianthera rupestris* (synonym *Casimirella rupestris* (Ducke) R.A.Howard) [35]
Common name	Mairá-potato

**Source**: [34].

**Table 3:** Botanical characterization of the Mairá-potato.

Species	*Casimirella ampla*	*Casimirella rupestris*
Plant	Rhizomatous shrub or vine with branches to 30 m climbing on trees, young branches somewhat angular, glabrous; leaves with petioles 8–10 mm long, glabrate, blade broadly lanceolate to elliptic, 8–20 × 3–10 cm, apex obtuse to acuminate, base nearly acute or rounded, central vein and veins prominent below	Scandent shrub, caudex tuber large and starchy; stems angular, stellate densely red–brown, pubescent
Leaf	Mature leaves glabrous or nearly so; pubescence of the inflorescence single-haired. Leaves 8–20 × 3–10 cm with petioles 8–10 mm long	Leaves with petioles 6–9 mm long, stellate pubescent; blade rhomboid to ovate, 10–15 × 6–8 cm, apex acuminate, base rounded, stellate pubescent above in central vein and veins.
Inflorescences	Inflorescence axillary or terminal, strigose	Inflorescence axis moderately star-shaped or tomentosa
Flowers	Flowers with calyx patelliform, lobes 1.3–1.6 mm long, lanceolate, densely hairy, petals oval–lanceolate oval, 3.5–4.3 × 1.4–2.0 mm, nearly equal, strigose outside, villous or tomentose or rarely crispate inside, apex inflexed, glabrous; filaments 2–3 mm, anther sacs globose, connective tapering to an apex extension, 0.6–0.8 mm; ovary glabrous, diam. 1 mm; hirsute; style 0.7 mm long, glabrous, slightly curved	Flowers with calyx 4 mm in diameter, lobes triangular–acute, 1.3 mm long, hirsute pubescent on the outside; petals oval–oblong, 4.1–4.3 × 1.6–1.9 mm, hirsute, villous inside, with a glabrate base, apex flexed; filaments glabrous, 2.5–2.6 mm long; ovary globose, diam. 1.2 mm hirsute.
Fruit	Globose to oblong, 7.5–8 × 3.8–4.0 cm, strigose inside the endocarp	Ovoid to globose drupe, 5 cm long, 4 cm in diameter, densely stellate pubescent, endocarp woody, smooth, to 0.7 mm thick, pubescent inside

**Table 4:** Structural formulas of phytochemicals isolated and identified in parts of the Mairá-potato plant.

Name of the Isolated/Identified Compound	Species	Technique of Analysis	Structural Formula of Compound	Ref.
Humiriantenolides A	*C. rupestris*	Thin layer chromatography (TLC) and nuclear magnetic resonance spectroscopy (NMR)	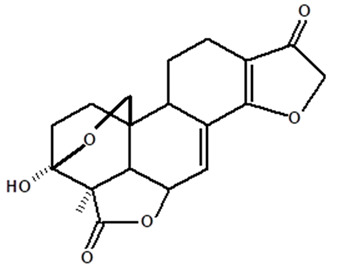	[13]
	*C. ampla*	Liquid chromatography		[14]
Humiriantenolides B	*C. rupestris*	Thin layer chromatography and nuclear magnetic resonance spectroscopy	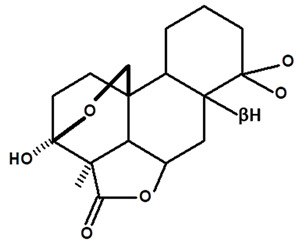	[13]
Humiriantenolides C	*C. rupestris*	Thin layer chromatography and nuclear magnetic resonance spectroscopy	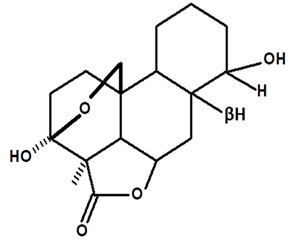	[13]
	*C. ampla*	Liquid chromatography		[14]
Humiriantenolides D	*C. rupestris*	Thin layer chromatography and nuclear magnetic resonance spectroscopy	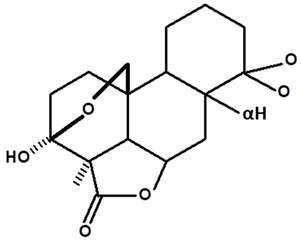	[13]
	*C. ampla*	Liquid chromatography		[14]
Humiriantenolides E	*C. rupestris*	Thin layer chromatography and nuclear magnetic resonance spectroscopy	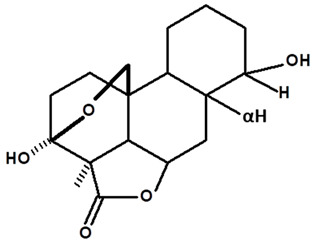	[13]
Humiriantenolides F	*C. rupestris*	Thin layer chromatography and nuclear magnetic resonance spectroscopy	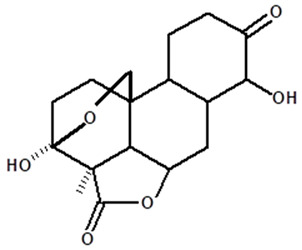	[13]
β-sitosterol	*C. rupestris*	Thin layer chromatography and nuclear magnetic resonance spectroscopy	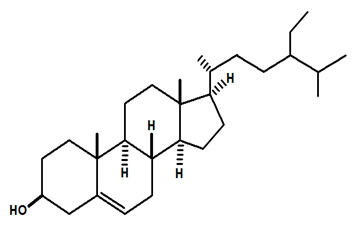	[13]
	*C. ampla*	Liquid chromatography		[14]
	*C. ampla*	Column chromatography and nuclear magnetic resonance spectroscopy		[20]
	*C. ampla*	Thin layer chromatography and nuclear magnetic resonance spectroscopy		[26]
Sodium thiocyanate	*C. ampla*	Liquid chromatography and atomic absorption spectroscopy	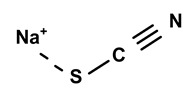	[15]
Potassium thiocyanate	*C. ampla*	Liquid chromatography and atomic absorption spectroscopy	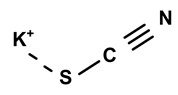	[15]
Sodium nitrite	*C. ampla*	Liquid chromatography and atomic absorption spectroscopy	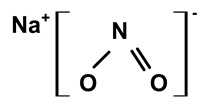	[15]
Potassium nitrite	*C. ampla*	Liquid chromatography and atomic absorption spectroscopy	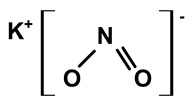	[15]
Sodium nitrate	*C. ampla*	Liquid chromatography and atomic absorption spectroscopy	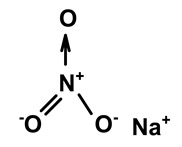	[15]
Potassium nitrate	*C. ampla*	Liquid chromatography and atomic absorption spectroscopy	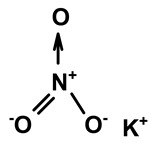	[15]
Lupeol	*C. ampla*	Gas chromatography coupled with a flame ionization detector (GC-FID)	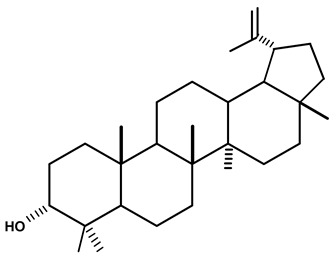	[19]
	*C. ampla*	Column chromatography and nuclear magnetic resonance spectroscopy		[20]
	*C. ampla*	Thin layer chromatography and nuclear magnetic resonance spectroscopy		[26]
β-sitosterol glycosylate	*C. ampla*	Gas chromatography coupled with a flame ionization detector	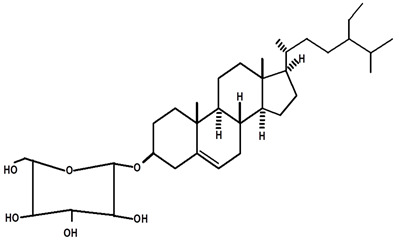	[19]
	*C. ampla*	Column chromatography and nuclear magnetic resonance spectroscopy		[20]
Annonalide	*C. ampla*	Gas chromatography coupled with a flame ionization detector	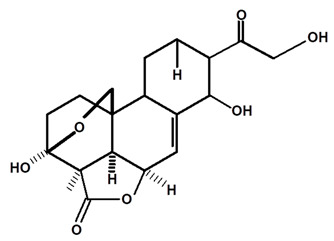	[19]
	*C. ampla*	Thin layer chromatography, nuclear magnetic resonance spectroscopy and infrared spectroscopy		[24]
	*C. ampla*	Column chromatography and nuclear magnetic resonance spectroscopy		[20]
	*C. ampla*	High efficiency liquid chromatography, nuclear magnetic resonance spectroscopy, and infrared spectroscopy		[25]
	*C. ampla*	Thin layer chromatography and nuclear magnetic resonance spectroscopy		[26]
Acrenol	*C. ampla*	Gas chromatography coupled with a flame ionization detector	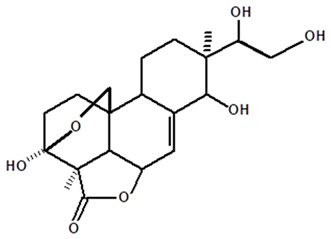	[19]
	*C. ampla*	Column chromatography and nuclear magnetic resonance spectroscopy		[20]
	*C. ampla*	Thin layer chromatography, nuclear magnetic resonance spectroscopy and infrared spectroscopy		[24]
	*C. ampla*	High efficiency liquid chromatography, nuclear magnetic resonance spectroscopy, and infrared spectroscopy		[25]
Humirianthol	*C. ampla*	Gas chromatography coupled with a flame ionization detector	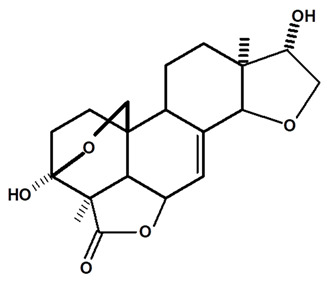	[19]
	*C. ampla*	Column chromatography and nuclear magnetic resonance spectroscopy		[20]
	*C. ampla*	Thin layer chromatography, nuclear magnetic resonance spectroscopy and infrared spectroscopy		[24]
	*C. ampla*	High efficiency liquid chromatography, nuclear magnetic resonance spectroscopy, and infrared spectroscopy		[25]
Phthalate	*C. ampla*	Column chromatography and nuclear magnetic resonance spectroscopy	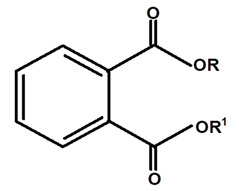	[20]
β-amyrin	*C. ampla*	Thin layer chromatography, nuclear magnetic resonance spectroscopy, and infrared spectroscopy	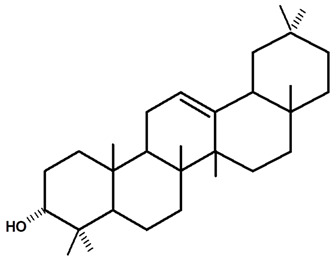	[24]
Glycosylated Plumerideum	*C. ampla*	Thin layer chromatography, nuclear magnetic resonance spectroscopy, and infrared spectroscopy	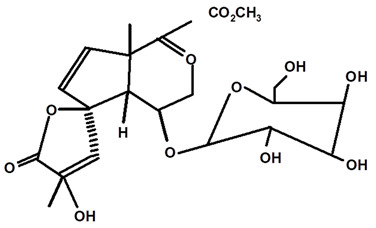	[24]
1βOβ-D glycopyranosyl plumeric	*C. ampla*	Thin layer chromatography, nuclear magnetic resonance spectroscopy, and infrared spectroscopy	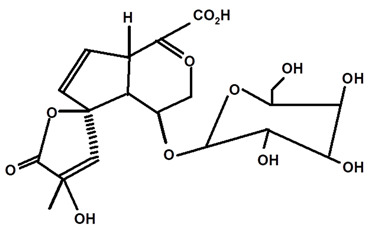	[24]
Acetylated Humirianthol	*C. ampla*	Gas chromatography coupled with a flame ionization detector and solvation with chloroform	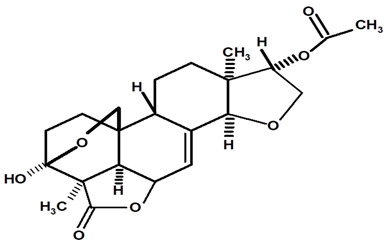	[22]
Acrenol diacetylate	*C. ampla*	Gas chromatography coupled with a flame ionization detector and thin layer chromatography	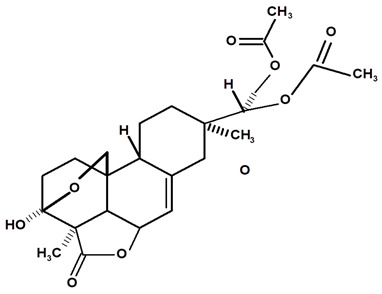	[23]
Icacinol	*C. ampla*	Gas chromatography coupled with a flame ionization detector and solvation with chloroform	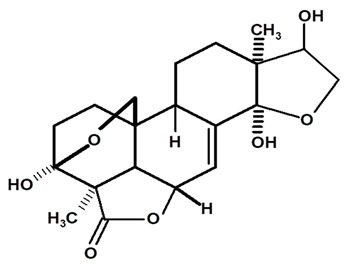	[22]
	*C. ampla*	High efficiency liquid chromatography, nuclear magnetic resonance spectroscopy, and infrared spectroscopy		[25]
Humirianthone	*C. ampla*	High efficiency liquid chromatography, nuclear magnetic resonance spectroscopy, and infrared spectroscopy	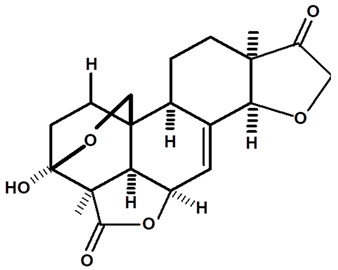	[25]
1-hydroxy-humirianthone	*C. ampla*	High efficiency liquid chromatography, nuclear magnetic resonance spectroscopy, and infrared spectroscopy	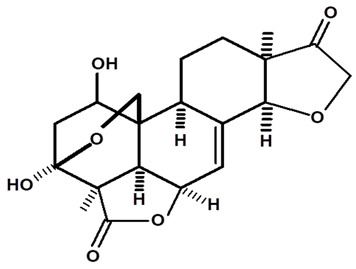	[25]
15R-humirianthol	*C. ampla*	High efficiency liquid chromatography, nuclear magnetic resonance spectroscopy, and infrared spectroscopy	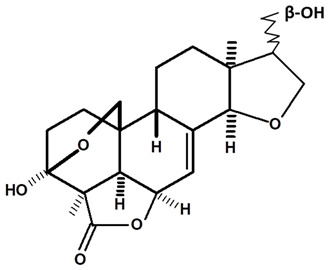	[25]
Patogonol	*C. ampla*	High efficiency liquid chromatography, nuclear magnetic resonance spectroscopy, and infrared spectroscopy	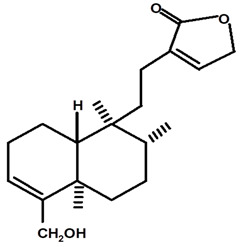	[25]
Patogonal	*C. ampla*	High efficiency liquid chromatography, nuclear magnetic resonance spectroscopy, and infrared spectroscopy	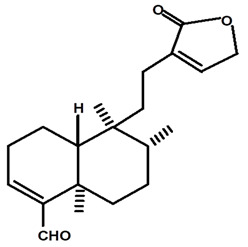	[25]
Stigmasterol	*C. ampla*	Thin layer chromatography and nuclear magnetic resonance spectroscopy	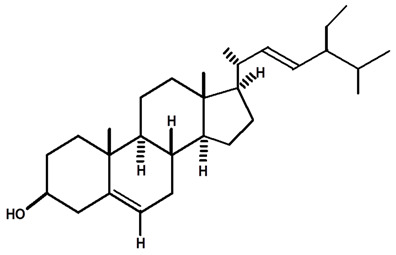	[26]
3-βOβ-D glycopyranosyl sitosterol	*C. ampla*	Thin layer chromatography and nuclear magnetic resonance spectroscopy	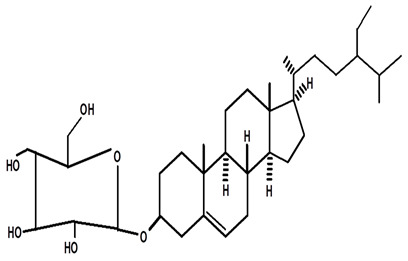	[26]

**Table 5:** Descriptor terms and Boolean operators used for searching the Google Academic, Scielo, Science Direct, Scopus, PubMed, and Web of Science databases.

Database	Descriptor Terms and Boolean Operators Used in the Search
Google Scholar	*Humirianthera ampla* AND *Humirianthera rupestris* *Humirianthera ampla* OR *Humirianthera rupestris*
Scielo	*Humirianthera ampla* AND *Humirianthera rupestris* *Humirianthera ampla* OR *Humirianthera rupestris*
Science Direct	*Humirianthera ampla* AND *Humirianthera rupestris* *Humirianthera ampla* OR *Humirianthera rupestris*
Scopus	ALL FIELDS = (*Humirianthera ampla* AND *Humirianthera rupestris*) ALL FIELDS = (*Humirianthera ampla* OR *Humirianthera rupestris*)
PubMed	*Humirianthera*
Web of Science	ALL = (*Humirianthera ampla* AND *Humirianthera rupestris*) ALL = (*Humirianthera ampla* OR *Humirianthera rupestris*)

## References

[1] FAO Food and Agriculture Organization of the United Nations (2014). International Fund for Agricultural Development. World Food Programme. The State of Food Insecurity in the World. Strengthening the Enabling Environment for Food Security and Nutrition.

[2] FAO (2022). Repurposing Food and Agricultural Policies to Make Healthy Diets More Affordable. The State of Food Security and Nutrition in the World 2022.

[3] Saath K.C.d.O., Fachinello A.L. (2018). Crescimento Da Demanda Mundial de Alimentos e Restrições Do Fator Terra No Brasil. Rev. Econ. e Sociol. Rural.

[4] Lebot V. (2009). Tropical Root and Tuber Crops: Cassava, Sweet Potato, Yams and Aroids. Crop Production Science in Horticulture.

[5] Pradeepika C., Selvakumar R., Nabi S.U., Sajeev M.S., Giri N.A. (2018). Ethnopharmacology and Toxicology of Threatened Tuberous Plant Genus *Ceropegia* sp. L.: A Review. Pharma Innov..

[6] Naczk M., Shahidi F. (2006). Phenolics in Cereals, Fruits and Vegetables: Occurrence, Extraction and Analysis. J. Pharm. Biomed. Anal..

[7] Chandrasekara A., Kumar T.J. (2016). Roots and Tuber Crops as Functional Foods: A Review on Phytochemical Constituents and Their Potential Health Benefits. Int. J. Food Sci..

[8] Ribeiro R.G. (2018). Estudo Etnobotânico e Físico-Químico Da Batata-Mairá (*Casimirella* spp.—Icacinaceae). Master’s Thesis.

[9] Kinupp V.F., Lorenzi H. (2014). Plantas Alimentícias Não Convencionais (PANC) No Brasil: Guia de Identificação, Aspectos Nutricionais e Receitas Ilustradas.

[10] Paschoal V., Souza N.S., Chaves D.F.S. (2015). Plantas Alimentícias Não Convencionais (PANC). Nutrição Clínica Funcional: Compostos Bioativos Dos Alimentos.

[11] Rizzini C.T., Mors W.B. (1976). Botânica Econômica Brasileira.

[12] Aparício M., Amoroso M., Mendes Dos Santos G. (2013). Os Suruwaha e Sua Rede de Relações. Uma Hipótese Sobre Localidades e Coletivos Arawa. Paisagens Ameríndias: Lugares, Circuitos e Modos de Vida Na Amazônia.

[13] Zoghbi M.D.G.B., Roque N.F., Gottlieb H.E. (1981). Humirianthenolides, New Degraded Diterpenoids from *Humirianthera* Rupestris. Phytochemistry.

[14] Zoghbi M.D.G.B., Roque Ν.F., Cabral J.A.S. (1983). Estudo Químico de *Humirianthera* Ampla (Miers) Baehni (Icacinaceae). Notas & Comunicações. Acta Amaz..

[15] Zoghbi M.d.G.B., Varejão M.d.J.C., Ribeiro M.N.d.S. (1988). A Presença de Substâncias Inorgânicas Tóxicas No Gênero *Humirianthera* (Icacinaceae). Acta Amaz..

[16] Varejão M.J.C., Ribeiro M.N.S., Zoghbi M.G.B. (1992). Variação Sazonal Dos Macro e Microelementos No Gênero *Humirianthera* (Icacinaceae), Em Função Da Idade. Acta Amaz..

[17] Varejão M.D.J.C., Zoghbi M.d.G.B., Diniz R.E.O., Ribeiro M.N.d.S. (1992). Variação Sazonal de Sulfato, Enxofre Total e Matéria Orgânica No Gênero *Humirianthera*, Em Função Da Idade. Acta Amaz..

[18] Howard R.A. (1992). A Revision of *Casimirella*, Including *Humirianthera* (Icacinaceae). Brittonia.

[19] Graebner I.B., Mostardeiro M.A., Ethur E.M., Burrow R.A., Dessoy E.C.S., Morel A.F. (2000). Diterpenoids from *Humirianthera* Ampla. Phytochemistry.

[20] Graebner I.B., Morel A.F., Burrow R.A., Mostardeiro M.A., Ethur E.M., Dessoy E.C.M., Scher A. (2002). Diterpenos Isolados de *Humirianthera* Ampla. Miers. Rev. Bras. Farmacogn..

[21] Monks N.R., Bordignon S.A.L., Ferraz A., Machado K.R., Faria D.H., Lopes R.M., Mondin C.A., De Souza I.C.C., Lima M.F.S., Da Rocha A.B. (2002). Anti-Tumour Screening of Brazilian Plants. Pharm. Biol..

[22] Burrow R.A., Morel A.F., Graebner I.B., Farrar D.H., Lough A.J. (2003). The Acetyl Derivative of Humirianthol. Acta Crystallogr. Sect. E Struct. Rep. Online.

[23] Burrow R.A., Morel A.F., Graebner I.B., Lough A.J., Farrar D.H. (2003). Absolute Configuration of Diacetylated Acrenol as Its Chloroform Solvate. Acta Crystallogr. Sect. E Struct. Rep. Online.

[24] Graebner I.B. (2003). Estudo Dos Constituintes Químicos Isolados de Plantas Medicinais Da Região Do Vale Do Purus No Acre (Amazônia) 133 f. Tese (Doutorado).

[25] Adou E., Williams R.B., Schilling J.K., Malone S., Meyer J., Wisse J.H., Frederik D., Koese D., Werkhoven M.C.M., Snipes C.E. (2005). Cytotoxic Diterpenoids from Two Lianas from the Suriname Rainforest. Bioorg. Med. Chem..

[26] Marques R.D.E.A. (2007). Estudo Fitoquímico e Biológico de *Humirianthera* Ampla Miers (ICACINACEAE). Master’s Thesis.

[27] Luiz A.P., Moura J.D.Á., Meotti F.C., Guginski G., Guimarães C.L.S., Azevedo M.S., Rodrigues A.L.S., Santos A.R.S. (2007). Antinociceptive Action of Ethanolic Extract Obtained from Roots of *Humirianthera* Ampla Miers. J. Ethnopharmacol..

[28] Strauch M.A., Tomaz M.A., Monteiro-Machado M., Ricardo H.D., Cons B.L., Fernandes F.F.A., El-Kik C.Z., Azevedo M.S., Melo P.A. (2013). Antiophidic Activity of the Extract of the Amazon Plant *Humirianthera* Ampla and Constituents. J. Ethnopharmacol..

[29] Marques R.A., Gomes A.O.C.V., de Brito M.V., dos Santos A.L.P., da Silva G.S., de Lima L.B., Nunes F.M., de Mattos M.C., de Oliveira F.C.E., do Ó Pessoa C. (2018). Annonalide and Derivatives: Semisynthesis, Cytotoxic Activities and Studies on Interaction of Annonalide with DNA. J. Photochem. Photobiol. B Biol..

[30] Amorim B.S., Cardozo N.D., Albuquerque P.M., Cabral F.N. (2020). Flora of the Ducke Reserve, Amazonas, Brazil: Icacinaceae. Rodriguésia.

[31] Amorim B.S. Casimirella. In Flora Do Brasil 2020 Em Construção. http://floradobrasil.jbrj.gov.br/reflora/floradobrasil/FB8022.

[32] Albuquerque M., Pinheiro E. (1970). Tuberosas Feculentas. Instituto de Pesquisas e Experimentação Agropecuárias Do Norte—IPEAN. Série Fitotec..

[33] Baehni C. (1936). Revision Des Genres Neoleretia, Mappia et *Humirianthera*. Candollea.

[34] GBIF G.B.I.F.—Acesso Livre e Aberto a Dados de Biodiversidade—Taxonomia. https://www.gbif.org/occurrence/search?dataset_key=7aeb3ded-7443-4512-95f7-2ec7ab0f308candtaxon_key=3596825.

[35] Amorim B.S., Stefano R.D. Icacinaceae. In: Flora Do Brasil 2020 Em Construção. http://reflora.jbrj.gov.br/reflora/floradobrasil/FB84155.

[36] Reflora-Herbário Virtual—Humirianthera Rupestris. http://floradobrasil.jbrj.gov.br/reflora/herbarioVirtual/ConsultaPublicoHVUC/ConsultaPublicoHVUC.do?idTestemunho=414989.

[37] Reflora-Herbário Virtual—Humirianthera Ampla. http://floradobrasil.jbrj.gov.br/reflora/herbarioVirtual/ConsultaPublicoHVUC/ConsultaPublicoHVUC.do?idTestemunho=5472396.

[38] Spruce R., Hooker J. (1851). Journal of a Voyage up the Amazon and Rio Negro. Hooker’s Journal of Botany and Kew Garden Miscellany.

[39] Balestra A.A. (2013). Tempos Mansos: História, Socialidade e Transformação No Juruá-Purus Indígena. Master’s Thesis.

[40] Hegnauer R. (1966). Icacinaceae. Chemotaxonomie Der Pflanzen. Lehrbücher Und Monographien Aus Dem Gebiete Der Exakten Wissenschaften (Chemische Reihe).

[41] Leitão-Barboza M.S., Kawa N.C., Junqueira A.B., Oyuela-Caycedo A. (2021). Open Air Laboratories: Amazonian Home Gardens as Sites of Experimentation, Collaboration, and Negotiation across Time. J. Anthropol. Archaeol..

[42] dos Santos G.M., Cangussu D., Furquim L.P., Watling J., Neves E.G. (2021). Indigenous Bread and Vegetable Pulp: Bonds between Past and Present in Indigenous Amazon. Bol. Do Mus. Para. Emilio GoeldiCiencias Humanas.

[43] Watling J., Shock M.P., Almeida G.Z.M.F.O., Kater T., De Oliveira P.E., Neves E.G. (2018). Direct Archaeological Evidence for Southwestern Amazonia as an Early Plant Domestication and Food Production Centre. PLoS ONE.

[44] Mostafa M., Nahar N., Mosihuzzaman M., Sokeng S.D., Fatima N., Rahman A.U., Choudhary M.I. (2006). Phosphodiesterase-I Inhibitor Quinovic Acid Glycosides from Bridelia Ndellensis. Nat. Prod. Res..

[45] Hernandez-Perez M., Rabanal R.M., De La Torre M.C., Rodriguez B. (1995). Analgesic, Anti-Inflammatory, Antipyretic and Haematological Effects of Aethiopinone, an o-Naphthoquinone Diterpenoid from Salvia Aethiopis Roots and Two Hemisynthetic Derivatives. Planta Med..

[46] Liu C.F., Lin N. (2006). Progress in Research on Mechanisms of Anti-Rheumatoid Arthritis of Triptolide. Zhongguo Zhong Yao Za Zhi.

[47] Laszczyk M.N. (2009). Pentacyclic Triterpenes of the Lupane, Oleanane and Ursane Group as Tools in Cancer Therapy. Planta Med..

[48] Sharma V., Kaur M., Sandhu K.S., Godara S.K. (2020). Effect of Cross-Linking on Physico-Chemical, Thermal, Pasting, in Vitro Digestibility and Film Forming Properties of Faba Bean (*Vicia faba* L.) Starch. Int. J. Biol. Macromol..

[49] Siddique H.R., Saleem M. (2011). Beneficial Health Effects of Lupeol Triterpene: A Review of Preclinical Studies. Life Sci..

[50] Salminen A., Lehtonen M., Suuronen T., Kaarniranta K., Huuskonen J. (2008). Terpenoids: Natural Inhibitors of NF-ΚB Signaling with Anti-Inflammatory and Anticancer Potential. Cell. Mol. Life Sci..

[51] Saleem M. (2009). Lupeol, a Novel Anti-Inflammatory and Anti-Cancer Dietary Triterpene. Cancer Lett..

[52] Shahlaei M., Ghanadian S.M., Ayatollahi A.M., Mesaik M.A., Abdalla O.M., Afsharypour S., Rabbani M. (2013). Molecular Modeling, Structure Activity Relationship and Immunomodulatory Properties of Some Lupeol Derivatives. Med. Chem. Res..

[53] Saleem M., Maddodi N., Zaid M.A., Khan N., Bin Hafeez B., Asim M., Suh Y., Yun J.M., Setaluri V., Mukhtar H. (2008). Lupeol Inhibits Growth of Highly Aggressive Human Metastatic Melanoma Cells in Vitro and in Vivo by Inducing Apoptosis. Clin. Cancer Res..

[54] Gallo M.B.C., Sarachine M.J. (2009). Biological Activities of Lupeol. Syst. Rev. Pharm..

[55] Fotie J., Bohle D.S., Leimanis M.L., Georges E., Rukunga G., Nkengfack A.E. (2006). Lupeol Long-Chain Fatty Acid Esters with Antimalarial Activity from Holarrhena Floribunda. J. Nat. Prod..

[56] Boakye Y.D., Groyer L., Heiss E.H. (2018). An Increased Autophagic Flux Contributes to the Anti-Inflammatory Potential of Urolithin A in Macrophages. Biochim. Biophys. Acta Gen. Subj..

[57] Saha S., Profumo E., Togna A.R., Riganò R., Saso L., Buttari B. (2020). Lupeol Counteracts the Proinflammatory Signalling Triggered in Macrophages by 7-Keto-Cholesterol: New Perspectives in the Therapy of Atherosclerosis. Oxid. Med. Cell. Longev..

[58] Sharma N., Palia P., Chaudhary A.S., Verma K.K. (2020). A Review on Pharmacological Activities of Lupeol and its Triterpene Derivatives. J. Drug Deliv. Ther..

[59] Colquhoun A.J., Venier N.A., Vandersluis A.D., Besla R., Sugar L.M., Kiss A., Fleshner N.E., Pollak M., Klotz L.H., Venkateswaran V. (2012). Metformin Enhances the Antiproliferative and Apoptotic Effect of Bicalutamide in Prostate Cancer. Prostate Cancer Prostatic Dis..

[60] Zachos G., Spandidos D.A. (1997). Expression of Ras Proto-Oncogenes: Regulation and Implications in the Development of Human Tumors. Crit. Rev. Oncol. Hematol..

[61] Jiménez-Escrig A., Santos-Hidalgo A.B., Saura-Calixto F. (2006). Common Sources and Estimated Intake of Plant Sterols in the Spanish Diet. J. Agric. Food Chem..

[62] Nair P.P., Turjman N., Kesie G., Calkins B., Goodman G.T., Davidovitz H., Ninmagadda G. (1984). Diet, Nutrition Intake, and Metabolism in Populations at High and Low Risk for Colon Cancer. Metabolism of Bile Acids. Am. J. Clin. Nutr..

[63] Gupta R., Sharma A.K., Dobhal M.P., Sharma M.C., Gupta R.S. (2011). Antidiabetic and Antioxidant Potential of β-Sitosterol in Streptozotocin-Induced Experimental Hyperglycemia. J. Diabetes.

[64] Shi C., Wu F., Xu J. (2013). Incorporation of β-Sitosterol into Mitochondrial Membrane Enhances Mitochondrial Function by Promoting Inner Mitochondrial Membrane Fluidity. J. Bioenerg. Biomembr..

[65] Ayaz M., Junaid M., Ullah F., Subhan F., Sadiq A., Ali G., Ovais M., Shahid M., Ahmad A., Wadood A. (2017). Anti-Alzheimer’s Studies on ß-Sitosterol Isolated from *Polygonum hydropiper* L.. Front. Pharmacol..

[66] Zhao M., Cheng J., Guo B., Duan J., Che C.T. (2018). Momilactone and Related Diterpenoids as Potential Agricultural Chemicals. J. Agric. Food Chem..

[67] Ramos D.V.B., Neto H.G., Azevedo M.S., Jardim I.S., Souza A.C.F., Cunha E.M.F., Marques E., Cunha F. (2009). Estudo Fitoquímico e Atividade Leishmanicida in Vitro de Extrato e Substâncias Isoladas de Humirianthera Ampla (MIERS). Proceedings of the Amazônia Ciência e Cultura. 61^a^ Reunião Anual da SBPC.

[68] Gonçalves H.P., Gonçalves-Neto H., Aprígio C.J.L., Azevedo M.S., Silva-Jardim I. QT54—Chemical study and evaluation of leishmanicidal activity from the ethanolic extract, eluate and isolated substance from Humirianthera ampla Miers. Quimioterapia- Chemotherapy. Proceedings of the SBPZ. Sociedade Brasileira de Protozoologia.

[69] Huggett A.C., Verschuren P.M. (1996). The Safety Assurance of Functional Foods. Nutr. Rev..

[70] OECD Organization for Economic Co-Operation and Development OECD Test Guidelines for Chemicals. https://www.oecd.org/chemicalsafety/testing/oecdguidelinesforthetestingofchemicals.htm.

[71] Barbosa J.R., de Carvalho Junior R.N. (2020). Occurrence and Possible Roles of Polysaccharides in Fungi and Their Influence on the Development of New Technologies. Carbohydr. Polym..

[72] Romero-Bastida C.A., Bello-Pérez L.A., García M.A., Martino M.N., Solorza-Feria J., Zaritzky N.E. (2005). Physicochemical and Microstructural Characterization of Films Prepared by Thermal and Cold Gelatinization from Non-Conventional Sources of Starches. Carbohydr. Polym..

[73] Haghighi H., Licciardello F., Fava P., Siesler H.W., Pulvirenti A. (2020). Recent Advances on Chitosan-Based Films for Sustainable Food Packaging Applications. Food Packag. Shelf Life.

[74] Valencia-Sullca C., Vargas M., Atarés L., Chiralt A. (2018). Thermoplastic Cassava Starch-Chitosan Bilayer Films Containing Essential Oils. Food Hydrocoll..

[75] Requena R., Vargas M., Chiralt A. (2018). Obtaining Antimicrobial Bilayer Starch and Polyester-Blend Films with Carvacrol. Food Hydrocoll..

[76] Sudheesh C., Sunooj K.V., Sasidharan A., Sabu S., Basheer A., Navaf M., Raghavender C., Sinha S.K., George J. (2020). Energetic Neutral N2 Atoms Treatment on the Kithul (Caryota Urens) Starch Biodegradable Film: Physico-Chemical Characterization. Food Hydrocoll..

[77] Jiang T., Duan Q., Zhu J., Liu H., Yu L. (2020). Starch-Based Biodegradable Materials: Challenges and Opportunities. Adv. Ind. Eng. Polym. Res..

[78] Pascoal A.M., Di-Medeiros M.C.B., Batista K.A., Leles M.I.G., Lião L.M., Fernandes K.F. (2013). Extraction and Chemical Characterization of Starch from S. Lycocarpum Fruits. Carbohydr. Polym..

[79] Tao K., Yu W., Prakash S., Gilbert R.G. (2019). High-Amylose Rice: Starch Molecular Structural Features Controlling Cooked Rice Texture and Preference. Carbohydr. Polym..

[80] Ojogbo E., Ogunsona E.O., Mekonnen T.H. (2020). Chemical and Physical Modifications of Starch for Renewable Polymeric Materials. Mater. Today Sustain..

[81] Silva D.A., Aires G.C.M., Pena R.d.S. (2020). Gums—Characteristics and Applications in the Food Industry. Innovation in the Food Sector Through the Valorization of Food and Agro-Food By-Products.

[82] Menzel C., González-Martínez C., Vilaplana F., Diretto G., Chiralt A. (2020). Incorporation of Natural Antioxidants from Rice Straw into Renewable Starch Films. Int. J. Biol. Macromol..

[83] Tapia M.S., Pérez E., Rodríguez P.E., Guzmán R., Ducamp-Collin M.N., Tran T., Rolland-Sabaté A. (2012). Some Properties of Starch and Starch Edible Films from Under-Utilized Roots and Tubers from the Venezuelan Amazons. J. Cell. Plast..

[84] Galindez A., Daza L.D., Homez-Jara A., Eim V.S., Váquiro H.A. (2019). Characterization of Ulluco Starch and Its Potential for Use in Edible Films Prepared at Low Drying Temperature. Carbohydr. Polym..

[85] Costa J.C.M., Miki K.S.L., Ramos A.d.S., Teixeira-Costa B.E. (2020). Development of Biodegradable Films Based on Purple Yam Starch/Chitosan for Food Application. Heliyon.

[86] Roy K., Thory R., Sinhmar A., Pathera A.K., Nain V. (2020). Development and Characterization of Nano Starch-Based Composite Films from Mung Bean (*Vigna Radiata*). Int. J. Biol. Macromol..

[87] Liporacci J.S.N., Mali S., Grossmann M.V.E. (2005). Efeito Do Método de Extração Na Composição Química e Nas Propriedades Funcionais Do Amido de Inhame (Dioscorea Alata). Semin. Ciências Agrárias.

[88] Baldwin E.A., Hagenmaier R., Bai J. (2011). Edible Coatings and Films to Improve Food Quality.

[89] Nakamura Y. (2015). Starch Metabolism and Structure.

[90] Zhu F. (2016). Structure, Properties, and Applications of Aroid Starch. Food Hydrocoll..

[91] Yao Désiré A., Charlemagne N., Degbeu Claver K., Fabrice Achille T., Marianne S. (2021). Starch-Based Edible Films of Improved Cassava Varieties Yavo and TMS Reinforced with Microcrystalline Cellulose. Heliyon.

[92] Niu X., Ma Q., Li S., Wang W., Ma Y., Zhao H., Sun J., Wang J. (2021). Preparation and Characterization of Biodegradable Composited Films Based on Potato Starch/Glycerol/Gelatin. J. Food Qual..

[93] Ye C., Chi H. (2018). A Review of Recent Progress in Drug and Protein Encapsulation: Approaches, Applications and Challenges. Mater. Sci. Eng. C.

[94] Ahmad S.U., Li B., Sun J., Arbab S., Dong Z., Cheng F., Zhou X., Mahfuz S., Zhang J. (2021). Recent Advances in Microencapsulation of Drugs for Veterinary Applications. J. Vet. Pharmacol. Ther..

[95] Lee S.Y., Ma J., Khoo T.S., Abdullah N., Nik Md Noordin Kahar N.N.F., Abdul Hamid Z.A., Mustapha M. (2021). Polysaccharide-Based Hydrogels for Microencapsulation of Stem Cells in Regenerative Medicine. Front. Bioeng. Biotechnol..

[96] Phillipson J.D. (2001). Phytochemistry and Medicinal Plants. Phytochemistry.

[97] Galanakis C.M. (2021). Functionality of Food Components and Emerging Technologies. Foods.

[98] Noore S., Rastogi N.K., O’Donnell C., Tiwari B. (2021). Novel Bioactive Extraction and Nano-Encapsulation. Encyclopedia.

[99] Alemzadeh I., Hajiabbas M., Pakzad H., Sajadi Dehkordi S., Vossoughi A. (2020). Encapsulation of Food Components and Bioactive Ingredients and Targeted Release. Int. J. Eng. Trans. A Basics.

[100] Chaudhary V., Thakur N., Kajla P., Thakur S., Punia S. (2021). Application of Encapsulation Technology in Edible Films: Carrier of Bioactive Compounds. Front. Sustain. Food Syst..

[101] Rostamabadi H., Falsafi S.R., Boostani S., Katouzian I., Rezaei A., Assadpour E., Jafari S.M. (2020). Design and Formulation of Nano/Micro-Encapsulated Natural Bioactive Compounds for Food Applications. Application of Nano/Microencapsulated Ingredients in Food Products.

[102] Favaro-Trindade C.S., de Matos Junior F.E., Okuro P.K., Dias-Ferreira J., Cano A., Severino P., Zielińska A., Souto E.B. (2021). Encapsulation of Active Pharmaceutical Ingredients in Lipid Micro/Nanoparticles for Oral Administration by Spray-Cooling. Pharmaceutics.

[103] Zhang J.Y., Pandya J.K., McClements D.J., Lu J., Kinchla A.J. (2021). Advancements in 3D Food Printing: A Comprehensive Overview of Properties and Opportunities. Crit. Rev. Food Sci. Nutr..

[104] Bird D.T., Ravindra N.M. (2021). Additive Manufacturing of Sensors for Military Monitoring Applications. Polymers.

[105] Mahmood M.A. (2021). 3D Printing in Drug Delivery and Biomedical Applications: A State-of-the-Art Review. Compounds.

[106] Agrawaal H., Thompson J.E. (2021). Additive Manufacturing (3D Printing) for Analytical Chemistry. Talanta Open.

[107] Jiang H., Zheng L., Zou Y., Tong Z., Han S., Wang S. (2019). 3D Food Printing: Main Components Selection by Considering Rheological Properties. Crit. Rev. Food Sci. Nutr..

[108] Wang M., Li D., Zang Z., Sun X., Tan H., Si X., Tian J., Teng W., Wang J., Liang Q. (2021). 3D Food Printing: Applications of Plant-Based Materials in Extrusion-Based Food Printing. Crit. Rev. Food Sci. Nutr..

[109] Singhal S., Rasane P., Kaur S., Garba U., Bankar A., Singh J., Gupta N. (2020). 3D Food Printing: Paving Way towards Novel Foods. An. Acad. Bras. Cienc..

[110] Yuan S., Shen F., Chua C.K., Zhou K. (2019). Polymeric Composites for Powder-Based Additive Manufacturing: Materials and Applications. Prog. Polym. Sci..

[111] Pérez B., Nykvist H., Brøgger A.F., Larsen M.B., Falkeborg M.F. (2019). Impact of Macronutrients Printability and 3D-Printer Parameters on 3D-Food Printing: A Review. Food Chem..

[112] Feng C., Zhang M., Bhandari B. (2019). Materials Properties of Printable Edible Inks and Printing Parameters Optimization during 3D Printing: A Review. Crit. Rev. Food Sci. Nutr..

[113] Dankar I., Pujolà M., El Omar F., Sepulcre F., Haddarah A. (2018). Impact of Mechanical and Microstructural Properties of Potato Puree-Food Additive Complexes on Extrusion-Based 3D Printing. Food Bioprocess Technol..

[114] Sun J., Peng Z., Zhou W., Fuh J.Y.H., Hong G.S., Chiu A. (2015). A Review on 3D Printing for Customized Food Fabrication. Procedia Manuf..

[115] Sun J., Zhou W., Huang D., Fuh J.Y.H., Hong G.S. (2015). An Overview of 3D Printing Technologies for Food Fabrication. Food Bioprocess Technol..

[116] He Y., Yang F., Zhao H., Gao Q., Xia B., Fu J. (2016). Research on the Printability of Hydrogels in 3D Bioprinting. Sci. Rep..

[117] Schwab A., Levato R., D’Este M., Piluso S., Eglin D., Malda J. (2020). Printability and Shape Fidelity of Bioinks in 3D Bioprinting. Chem. Rev..

[118] Ma Y., Schutyser M.A.I., Boom R.M., Zhang L. (2021). Predicting the Extrudability of Complex Food Materials during 3D Printing Based on Image Analysis and Gray-Box Data-Driven Modelling. Innov. Food Sci. Emerg. Technol..

[119] Liu Z., Zhang M., Bhandari B., Wang Y. (2017). 3D Printing: Printing Precision and Application in Food Sector. Trends Food Sci. Technol..

[120] Liu Z., Bhandari B., Prakash S., Mantihal S., Zhang M. (2019). Linking Rheology and Printability of a Multicomponent Gel System of Carrageenan-Xanthan-Starch in Extrusion Based Additive Manufacturing. Food Hydrocoll..

[121] Moher D., Liberati A., Tetzlaff J., Altman D.G., Altman D., Antes G., Atkins D., Barbour V., Barrowman N., Berlin J.A. (2009). Preferred Reporting Items for Systematic Reviews and Meta-Analyses: The PRISMA Statement. PLoS Med..

